# Address of W. H. Goddard, Delivered before the Miss. Val. Ass. of Dental Surgeons, Feb. 33, 1855

**Published:** 1855-04

**Authors:** 


### Dental Register of the West

VOL. VIII. APRIL, 1855. NO. 3.

### Original Communications.

### ADDRESS OF DR. W. H. GODDARD.

DELIVERED BEFORE THE MISSISSIPPI VALLEY ASSOCIATION OF DENTAL SURGEONS, FEB. 23D, 1855

GENTLEMEN OF THE M. V. A. OF DENTAL SURGEONS:

*Friends and Associates*:—Again, in the order of a permissive Providence, that Providence which watches over all good, and scans all evil, the annual cycle, has brought us together. In rising to address you, I cannot forbear the expression of regret, that a sense of my own inadequacy to the task I have assumed, forbids the hope, that I shall be able either to answer *my* desires, or *your* expectations. Notwithstanding, I will enter upon the effort, trusting that your liberal intelligence, will supply my deficiency, entreating you in the language of one of old, that if not for myself, at least you will "*hear me for my cause, and be silent that you may hear.*"

The vicissitudes, the changes,—fortunes which the world has felt and looked upon, in the last year, though perhaps interesting, will not be necessary to notice, on this occasion.

They are but the history, to a larger or less extent, of every other year, from the beginning till now. For as *days* in their ordinary development and progress, bear a family likeness to each other, so also of years; each following in the other's footsteps, and all hastening with their time gathered burdens, to that vast "trysting" ground of all earthly incidents, the measureless eternity of God. If it be a true thought, that *Time*, in unfolding process from the great wheel of immortal destiny, receives with daguerrian exactness, the universal impress of mind, bearing with continuous passage, from this to the other world, the witness of things done in the flesh, then needeth it not that divine philosophy shall tell, that "man is fearfully and wonderfully made." But if fearfully, also sublimely. There is a moral grandeur in the idea, that the entire book of Time, shall be preserved in eternity, to be read of all, and read forever; where each immortal, conscious of what *he is*, may behold, as in an antecedent mirror, the shadowings of what *he was*, and there make his own contrast. It is great, it is like God. Impressed with this truth, one has said with emphasis,

> "O memory, memory, thou good man's friend,
> Thou sinners bitter curse ;'"—

The importance, however, of a just course in life, is not alone to be gathered from the facts and fears of a future state of existence; but also from the immediate sequences, which follow good and evil, in this world.

The good acts of mankind, do not await, the mortal passage before they receive reward, there is a prelibation, even here; so also of the bad. The pre-monitions of retribution, work in the heart of evil, while yet in the estate of earth.

But to our subject more particularly. This is, *The Philosophy of Association.* First considered, in its general development, in its origin and in its facts.

The history of the human race, is but a practical commentary, upon the laws and obligations of Association. The embodiment of a great principle, is therein exhibited to the world whose original inception, and first communication, were of God himself, when he said, "let us make man." To this point may be traced, the birth of this cause, whose amplification and continuance, have since entered into all the ramifications and diversities of intelligent society. From the same original, it descended also, to become a law of nature, and is found to exist, in every possible form of material combination, from the grain of sand we tread upon, to the golden Sun, which lights us on our way.

While then, as neeessary to scientific research, and beneficial promulgation, *association* may be regarded simply as an attribute or instrument of human progression, in a more comprehensive and enlarged view of the subject it is clearly the law of God. By him appointed, as a principle, it pervades all nature; from him imitated as an instrument, it reaches to all purpose. Variety in unity, is the manner of its developments, while in its subjective manifestations, "its name is legion."

That this is one of the Divine arrangements, which is more a blessing to the world than others, will not be doubted by any, who have at all considered the subject.

In the kingdom of mind, every intellectual process, every deduction, whether simple or compound, plain or abstruse, must stand indebted, for a cause to ideal association. This is the parent of both thought and reflection, and tends to meditation, (which one of the philosophers denominates "the first class of study,") all its powers whether pleasing or profitable.

The same thing obtains, also, in morals and in physics. The great liberty maxim, in "union there is strength," is as applicable to this subject, as to that which constituted its procuring cause. Union and fraternity in *association*, when applied to useful knowledge, whether natural or scientific, congressively calls together, and consolidates in munificent unity, the merits and mind of every age, as well as of every nation.

This is earthly immortality, and not unworthy of human pursuit. Great men live in their works. In this respect, Aristotle and Esculapius, with every other antecedent magnet of truth and science, are intellectually as much the associates of the present time, as though they were still the living lights and oracles of the Grecian schools.

When will the thunders of Demosthenes cease to roll, or Plato's sun go down? In this regard, every association having for its object and aim, mental and scientific culture and promulgation, presents a philosophic congress of ages, in which the discoveries and improvements of all times, and all nations past, in order to their beneficial enlargement, and permanent expansion, are made to pass in review, before the accumulated light of present intelligence.

This is a Divine arrangement, and its heavenly philosophy which is clearly apparent, emanates from a higher power. Were it not for this, the world, so far as improvement, or advancement might be considered, would stand still. Each successive age, in dissociate isolation, would receive no advantage from antecedent discovery, but would be compelled to travel over the same ground of its predecessor, from the beginning to the end, in order to arrive at the same knowledge. By consequence, the entire journey of human progress and intellectuality, would be circumscribed within the limits of the first link, only, of what now constitutes a magnificent chain of scientific sequences, along the whole line of which, the improved mind, passes and re-passes, with electro magnetic speed. In this, may be discovered, what is the true relation and bond work of association.

The same principle applies to persons, as well as to things. As the kernel of wheat must be mashed by the grinder, before the generous flour can be obtained for use, so the shell of individualism must be crushed, in the mill of association, before the inherent good properties of our common nature can be made to appeal. Such is, or at least seems to be the Divine will, concerning the human family.

The light of this philosophy, shows clearly the past, to be the inheritance of the present, and the pledge of the future. The present in this respect, reaches back and gathers from the past, all the benefits of antecedent experience and research. This is its storehouse, its magazine of knowledge. But the appropriation brings with it also an obligation. For just so far as it takes to itself those benefits, it pledges itself to the future, constructively at least, not to neglect and abuse, but use and improve, and faithfully hand forward to the next generation, the undiminished capital received, and with interest added. The Bible tells us that "no man liveth to himself," which is as true in science as it is in divinity.

More especially is this the case in that science, or philosophy, which purposes as its great aim, to alleviate suffering and remove pain.

In its nature, science is not secret, but associative, and largely deals in compounds and combination.

Whatever is made secret, in pretended scientific discovery, by such process, subjects itself necessarily to popular distrust.

The peculiarity of nostrum mongers, those naturalized poisoners of the human family, is, their remedies are all mysteriously secret. But like the *Thug* of Asia, their secresy is exceedingly death-like, it kills in the dark. There are exceptions, doubtless, to this rule; but these are most unfortunate, for they will in spite themselves wear the *counterfeit* in popular estimation, though their development exhibit in reality, the forehead of truth. A fact discovered, when examined and proclaimed by associative wisdom, is at once legalized and accepted in the public mind. It takes a ready, and undisputed rank, which unassisted merit would be a long time in achieving. Perhaps would die, ere its object was gained.

There have been such in almost every profession, whose talents and usefulness, association would have brought out, but it was not at hand, and they rested upon the lap of society, like the poets flower,

> "Born to blush unseen,
> And waste its sweetness on the desert air."

This brings us to the second part of our subject. Associaion, in its objects and influences.

Nothing can take place in the universe of God, without the entertainment of both an object and an influence.

Paiticularly is this the case, where two or more persons, or principles, are consolidated in Association. These may be, for either good or evil, according as the purpose may be expressed, in the bond or pledge of organism. Evil associations, as well as good ones, may, and doubtless *do* exist in the world, whose covering is the cloak of Erebus, whose doings are the deeds of devils, and upon whose conclaves of iniquity, truth and virtue would blush to look. But, so far from this being an argument against the propriety of association, it is the best plea, to be urged in its defence.

The base counterfeit is always prima facia evidence of the existence of the genuine coin. The great object of association is set forth, in what is understood to have been the procuring cause of the organization of human society, and the establishment of human laws. In the beginning, when nought but individualism reigned, *might* was *right*, and the law which governed was the law of combat. The law of the spear and sword, of the strong against the weak. But for the better protection of the whole, which reason suggested, ought to be guaranteed in some way by common consent, each individual submitted to his peers, his personal prerogative to judge and act for himself in matters of contest; the sword was placed in the hands of impartial justice, and legal association begotten of necessity, was born of equal rights.

Like Minerva from the brain of Jupiter, the organic principle came forth from the womb of cause, armed and equipped with sword and balance, to take the judgment seat. The object was equal protection to each and all, guaranteed by the consolidated power of the whole. Scientific associations of every kind and character, are subsidiary to this. They revolve as inner circles of this vast wheel of outer circumference, and light up its horizon as stars light up the firmament, reflecting upon the dark and unorganized masses, the brightness of discovery and the benefits of progressive knowledge. Their object, though the lesser in the scale is like the larger, to enlighten mankind by bringing together, into a common depository the rich results of scientific investigation.'

This principle has led to the building up of institutions of various grade and character, for the promotion of science throughout the world,—an illustrious example of which, is the great Smithsonian Institute of our own country. To say that the object has been fully attained, would be to declare the battle of life, to be fought and won ; this is not the case, though much has been *done*, more remains to *do* ; "there is much land yet to be possessed." And this is the work of association, whose benefits to scientific progress are not to be calculated.

What the circulation of the blood is to the human system, the principle of association is to society, it is the motive life and power of its organic being.

The influence of association, quickens the intellect and gives point to the qualities of mind. It lends to logic its solidity, and plumes the wings of the rhetorician for his exuberant flights and figures of fancy.

And though some perhaps, coming too near the light and heat of truth, may be hurled, Icarus like, from their too presumptuous and daring height, their fall from the ambitious eminence they toiled to attain, without the legitimate helps of knowledge, must be but a simple demonstration of the fact—that the waxy wings of ignorance, will not do to soar upon.

Association is also the touch-stone and fire of truth. It brightens and strengthens the real steel, but melts and destroys the hypocritical dross. It detects falsehood and exposes ignorance. Its requirement is, that every member shall be able to pronounce the scientific and associative "Shibboleth," the result of which would be to rid society of quacks and pretenders ; to give a ready currency to the true coin, and consign the counterfeit to an ignominious and merited oblivion.

We are now brought to consider this doctrine as applied to ourselves, to our fraternity. In addressing you, as an Association of dentists, you will not accuse me of detracting from the legal knowledge of the profession, when I assert, that Dental Surgery is yet in its infancy. That this is true both as regards its nature and its practice, together with its claim to high appreciation, will not be doubted by any whose opinion is valuable, with intelligent and cultivated minds. Although much has been done, to secure its acceptance and elevation, in the public mind, as well as for its more perfect culture in college halls, much remains yet to accomplish before the science will be able to take its rightful place among the more important and useful of the learned profession. This is the rank and position we assume for it, which the influences of association will abundantly secure.

Without going into an elaborate argument to prove that Dentistry is the highest degree in the "Scalpel" of knighthood, which we will presume, for the sake of our cause is taken for granted, we will remark, that the exceeding delicacy of the practice, if there was no other reason, would lead inevitably to this conclusion. Though a cognate of the great medical structure, which like surgery, not many years since was embraced in the course of the student, there is no branch of the parent profession, which so eminently depends for success upon a highly polished and delicate sensibility in its pursuit, as this science. That every Dentist, therefore, if he would prosper in his caling, should study to become a finished gentleman, is too obvious to need an assertion.

This necessity, has to a great extent, grown up with its distinctness as a science. In the days of the old fashioned Pullikin, and other ruder instruments, in the hands of blacksmiths and barbers, this was not so much the case.

The kingdom of truth then, to a large extent at least, like the unbroken wilderness lay unexplored. With the clearing has come the culture, which has introduced another philosophy in Dental Science. A philosophy, which if universally adhered to and carried out, will soon demonstrate not only that Dental Practice is most profitable and useful, but at th same time, distinguished and honorable.

A high state of moral and mental refinement is therefore absolutely necessary, as a pre-requisite to usefulness and distinction, in the prosecution of this science.

The necessity for this sort of qualification, which may appear singular to some, may be attributed to the fact, that in all the higher walks of the professon, the practice falls with the cultivated and the polite of society, and to a large extent among females. With such, a rude behavior, and rough exterior, no matter how scientifically erudite and learned the practitioner might be, he would meet with but little encouragement.

The use of anasthetic compounds also, should be always refused the patient, unless in presence of a third person, and that person, either a physician or one of the same sex with the patient.

It is not necessary to advert here to the dreadful calamity, that has recently followed the indiscreet use of these; in which I believe, the reputation of an innocent man has been blasted and the profession falsely dishonored; the case of the unfortunate Beale is a warning to all. If chloroform, or any kindred compound is desired, both the character of the operator and the profession demand, that the administration should be in the presence of third parties.

Next to polite accomplishments, and right views, as connected with the *manner* of the profession, and in many respects a desideratum, not only in this, but in every other honest vocation of life, is an unimpeachable moral reputation. If Christian faith be added, so much the better. Moral character is so obviously demanded, to constitute the basis of public confidence and trust, without which no calling can hope to succeed, it is hardly necessary to offer an elaboration of the subject. And yet, because there are some, learned too, who in their over-earnest pursuit of other things, neglect the culture of moral principle altogether, a word or two on the subject may not be out of place.

Next to Christianity, which is admitted by all to be the great light of the world, and which, in social ethics, is what the soul is to the body, the life thereof; refinement in morals, is the chief element of excellence in all practical life. No matter how learned, no matter how scientific, no matter how worthy in every other particular a man may be, if he be defective in morals, he will labor in vain, for either profit or distinction. The highest degree in the scale, as has already been hinted is Christianity. To become a Christian however, embraces a mightier obligation, and a superior power, than ought which may be claimed by science. Still it is the best step for a professional man, simply considered as such, leaving out of the scale all higher motive than professional success. But while this high and holy position, *may be* dispensed with, and success still attained unto, it is not so with moral reputation. That must not be disregarded if the practitioner would elevate himself and his calling. Especially is this the case with us. Moral excellence, in the Dental Surgeon, is the fulcrum of the Archemedian lever of science, which is to lift both the professor and the profession, into their rightful seat of meritorious estimation. It is greatly to be regretted, that this principle has not been so universally regarded as its importance demands. A strict adherence to its rules, would have saved the fair fame of some, and prevented the impeachment, doubtless, of many. The nature of the practice renders it of the first importance, that the Dentist should be like Cæsar's wife, not only innocent, but unsuspected.

We feel that we can not do better than earnestly to urge upon the attention of the association the importance of this subject. It is true, what we have said, may be regarded as among the relative, rather than the positive elements of our association; but be it remembered that *relation*, in ethical philosophy, is no less important than *position*.

*Scientific ability*, which is the great center of our system, as well as the object of our association, must now claim our attention for a moment. This of course, is the foundation of the entire structure. Without it, all the rest, however excellent in themselves, would be valueless as attributes of professional association.

Professional excellence is the parent of both fame and fortune, and is therefore a noble object for human ambition to aim at. The love of fame, has been the procuring cause of greater energy, sacrifice and hazard, on the part of man, than all other causes combined. It has robbed the battle of its brutal fears, cloaked the cannon in its angry roar, and led the soldier, fearless, to pluck bright honor from the bayonets reeking point. As a spirit of omnipotence, under its inspiration, the thunders of the Forum roll their echo's from Demosthenes, to the modern Daniel; while in obedience to the same power, the earthquake of arms, ranges, from Marathon to Waterloo, and from Waterloo to Buena Vista. So also in the fields of science. From Copernicus to Newton, the spirit flies, and from Newton to Franklin, who, touched by the inspiration, commands the thunder from his cloudy car, and he lays his sceptre harmless at the Philosopher's feet.

Profundity in scientific worth should be the great object in professional pursuit. But in the race for fame, great caution should be exercised, lest desire out-run desert. In the present age of haste, when all the world seems in a hurry for something, it knows not what, when even, the spark upon the wires, is rebuked by impatience, for untelegraphic sluggishness, it is not strange that the world should be filled with quacks and quackeries of every sort. Young America, in politics, has proved the fruitful parentage, not only of graduated M.D.'s in petticoats, but of moral mendicants, and medicinal nostrum-mongers, who like the yellow covers of literary bastardy and corruption, fill the land. This whole thing should be repudiated and avoided in dental science, as the murrain among the cattle or the mill-dew upon the wheat.

Don't be in a hurry gentlemen, there is time enough yet. The "hasty plate of soup" system, which grinds out graduates, "secundum artem," as the yankees are said to make nutmegs, and pumpkin seeds, and sometimes too from material about as promising of fruitful growth, is not only a dishonor to the profession, but a daring danger to community.

Whence arises, we may ask, the most reasonable objection, that is urged against the dental profession, viz., that it is an unsafe practice, and that the operator is as likely to do evil as good. The answer is, because of unscientific and illegal practitioners. Were it not, that impostors impose themselves upon the public, men of little sense and less science, who know about as much of the profession they pretend to practice, as they do of the inhabitants of the moon, this state of things would not be. Because of a lack of legal knowledge in the public mind to detect them, short of dear bought experience, they are able to practice their impositions unchecked. The consequence is, not *their* disparagement, but the discredit of the profession. It is, or ought to be, the special business and duty of our association to protect the public from such impostors, and by doing so, it will also protect itself. If it be asked how this is to be done, the answer is, let none go forth, patented with institutional privileges, under diploma or certificate, who are not well qualified for the work they have undertaken.

Much of this evil is attributable to the profession itself. It does not sufficiently respect its own calling. A lack of self-respect, is not unfrequently the cause of disrespect from others. More especially when the philosophy of the subject is not perfectly understood. Such is, at this time, eminently the case with dental surgery. And such is likely to remain the case, until the profession shall be sufficiently well-understood, to speak for itself, in the manner of its practice.

When this shall be the case, when, as in many other professions, the public shall be educated, to know the true dentist by his skill and manner, in which he manipulates a time devoutly to be wished for, then impostors will cease to exist. Then, while the counterfeit is being nailed to the counter of opprobrium, by the hand of legal science, the profession will rise into its proper place. Till then, it must be guarded by association.

Based upon the erect integrity of scientific and moral ability, it should stand as the pillar of Hercules, resistent of all detraction, and repellant of every foe.

Dental education, of which we have as yet said nothing, taking it for granted, that the judgment of the association is well informed on that subject, should be thorough. No considerable success could be anticipated, with an imperfect education. This should comprise, in the scientific department, all that is taught in our dental colleges; add to these, the moral also, conscientiousness, candor, honesty, morality, and gentlemanly urbanity. Thus furnished and qualified with a perfect knowledge of this particular science, and the man is fitted for his work. There is another point which we should not pass without notice; it is equality of estimate and treatment of all patients. As science takes no cognizance of persons, because of either their high or low estate, so neither should the adminstration of science, even though he run the risk, in some instances, of working for naught. There is nothing more characteristic of a circumscribed intellect, and defective moral sense, than for a professor of any liberal science, to make a prejudicial distinction in the application of his calling, betwixt the poor and the rich. This is not only wrong "per se," but may work greatly to the disadvantage of the profession. The French king, who always said to the beggar, who asked of him an alms, "Remember me when you wear the crown," affords a significant commentary upon this principle.

The poor of to-day, may be the rich of to-morrow; a fair equivalent, and no more should be asked for. But time will not permit me to make extended remarks upon the subject of fees for professional services. I will only add a few words upon the subject.

"The practice of under-charging, as a means of professional advancement, involves an unworthy motive, and the effect is to lower the dignity of the profession. But by no means, are the principles of Dental Ethics opposed to the exercise of humanity and generosity in rendering gratuitously our services when deemed necessary; on the contrary, regard for the character of the profession is to be added to the dictates of humanity and generosity, as an incentive for extending to the poor, the resources of our art, and in graduating the amount of pecuniary obligation by the ability of patients.

The dentist is at liberty to place such an estimate on the value of his knowledge and skill, as he may conscientiously think justice to himself demands. But there is no professional justification in levying upon the rich, to make up the deficiency of a gratuity to the poor. Neither should the poor ever be suffered to leave your offices, in pain, because they have no money.

By a course like this, a high reputation will be more easily gained than by any other, which in the estimation of all good men, is better than gold, "Yea, than much fine gold." There is one point more, and it is the last I shall glance at on this occasion.

It is the duty which professors owe to each other. Almost every liberal profession is viewed by the public eye as a whole, and each member is recognized as a representative of the entire body. This being the case, there is as much necessity for harmony in order to the healthful progress of the cause as for the peace of individuals.

It would be ridiculous for the eye to find fault with the mouth, or the hand to fight with the foot. In either case, not those members alone, but the whole body would be made to suffer. So, also, of this profession. The bearing therefore of practitioners should always be urbane, kind and gentlemany towards each other. If this is not always the case, it is because of some of the defects heretofore named in this paper. As all professions then are consolidate, to a greater or less extent, in the public mind and each individual member held responsible for the whole, it is obviously suicidal, to fall out by the way, and not only so, but fratercidal also. The result is, that by construction, every member of the association is constituted a guardian of the common honor; and it is his duty faithfully to discharge his trust. To this end never speak disparingly of the talents or work of another, never suffer the profession to be abused in your presence, nor take liberties with it yourself; anonymous letters and pamphlets, which sometimes appear, always consider the handy-work of cowards and assassins, and treat them as such. For he who would abuse you, from behind the cloak of incognito, would not hesitate also to stab in the dark.

First be *men*, then, if you will take our advice, be Christians also; Christians in the fullest sense of the word; in accordance with the sentiments of the greatest man of modern days—Daniel Webster, the immortal Sage of Marshfield, who near the close of his life, speaking in the language he had before used, on the death of a valued friend, whose experience had been like his own, said, "Political eminence and professional fame fade away and die with all things earthly. Nothing of character is really permanent but virtue and personal worth. These remain. Whatever of excellence is wrought into the soul itself belongs to both worlds. Real goodness does not attach itself merely to this life; it points to another world. Political or professional reputation can not last forever; but a conscience void of offence before God and man is an inheritance for eternity. *Religion*, therefore, is a necessary and indispensable element in any great human character.

*There is no living without it.* Religion is the tie which connects man with his creator, and holds him to his throne. If that tie be all sundered, all broken, he floats away, a worthless atom in the universe; its proper attractions all gone, its destiny thwarted, and its whole future nothing but darkness, desolation and death.

A man with no sense of religious duty, is he whom the Scriptures describe in such terse but terrific language, as living "without God in the world." Such a man is out of his proper being, out of the circle of all his duties, out of the circle of all his happiness, and away, far, far away, from the purposes of his creation."

